# Angiogenesis and Apoptosis: Data Comparison of Similar Microenvironments in the Corpus Luteum and Tumors

**DOI:** 10.3390/ani14071118

**Published:** 2024-04-06

**Authors:** Taehee Min, Sang-Hee Lee, Seunghyung Lee

**Affiliations:** College of Animal Life Sciences, Kangwon National University, Chuncheon 24341, Republic of Korea

**Keywords:** ovary, corpus luteum, tumor, angiogenesis, apoptosis

## Abstract

**Simple Summary:**

Angiogenesis and apoptosis, which contribute to tissue formation and regression, play a critical role in the corpus luteum and tumors. These processes involve angiogenesis- and apoptosis-related factors, including hormones, growth factors, extracellular factors, cytotoxic factors, and cytokines. Thus, we reviewed similar mechanisms regarding the physiological events of the corpus luteum and tumors in animals. Further, we suggested a novel research animal model for finding animal disease mechanisms in the ovary.

**Abstract:**

The corpus luteum is a temporary endocrine gland formed in the ovary after ovulation, and it plays a critical role in animal reproductive processes. Tumors rely on the development of an adequate blood supply to ensure the delivery of nutrients and oxygen and the removal of waste products. While angiogenesis occurs in various physiological and pathological contexts, the corpus luteum and tumors share similarities in terms of the signaling pathways that promote angiogenesis. In the corpus luteum and tumors, apoptosis plays a crucial role in controlling cell numbers and ensuring proper tissue development and function. Interestingly, there are similarities between the apoptotic-regulated signaling pathways involved in apoptosis in the corpus luteum and tumors. However, the regulation of apoptosis in both can differ due to their distinct physiological and pathological characteristics. Thus, we reviewed the biological events of the corpus luteum and tumors in similar microenvironments of angiogenesis and apoptosis.

## 1. Introduction

**Figure 1:**
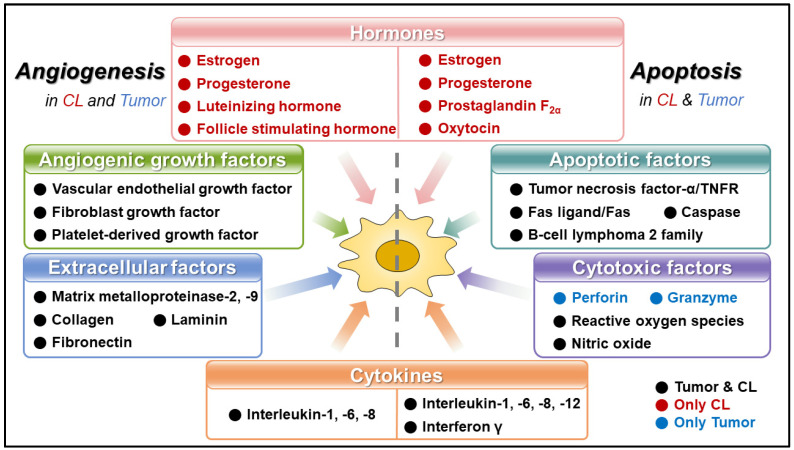
Angiogenesis- and apoptosis-related factors in the ovarian corpus luteum and tumor. In the corpus luteum and tumors, hormones, angiogenic growth, extracellular, cytokines, and apoptotic and cytotoxic factors contribute to angiogenesis and apoptosis. Black letters: tumor- and CL-regulated; red letters: CL-regulated; blue letters: tumor-regulated; CL: corpus luteum.

Angiogenesis and apoptosis are two fundamental biological processes that play crucial roles in the development, maintenance, and functioning of various tissues and organs in humans and animals. These processes are also intimately involved in the pathophysiology of diseases, including ovaries and cancer [1,2,3,4]. The microenvironments of angiogenesis and apoptosis in the corpus lutem and tumors share some similarities (Figure 1).

The angiogenic microenvironment is characterized by a complex interplay of pro-angiogenic and anti-angiogenic factors, signaling molecules, and cellular components. In both the corpus luteum and tumors, angiogenesis is initiated by a state of hypoxia, which releases pro-angiogenic factors that stimulate the formation of new blood vessels [2,5]. Also, the angiogenic microenvironment involves cellular components, including endothelial cells, pericytes, and immune cells. Endothelial cells play a central role in angiogenesis, forming the inner lining of blood vessels. Pericytes, which surround the endothelial cells, provide stability and regulate vessel maturation. Immune cells, such as macrophages and lymphocytes, can promote or inhibit angiogenesis depending on their phenotype and secreted factors [1,6].

Apoptosis, or programmed cell death, is a tightly regulated process that is essential for maintaining tissue homeostasis. Also, the microenvironment of apoptosis involves a complex network of signaling molecules, cellular components, and extracellular factors [7]. In the corpus luteum and tumors, apoptosis is regulated to maintain tissue homeostasis and eliminate abnormal or unwanted cells [8,9]. The microenvironments of apoptosis in the corpus lutem and tumors share several similarities. These similarities involve the cellular interactions, signaling molecules, and extracellular factors that govern the apoptotic process.

Therefore, we reviewed the similar microenvironments and mechanisms of angiogenesis and apoptosis in the corpus luteum and tumors. Understanding these shared features can provide insights into the regulation and dysregulation of angiogenesis and apoptosis in both microenvironments, allowing for an animal research topic to be approached from a novel perspective.

## 2. Angiogenesis in Corpus Luteum and Tumor

Angiogenesis is a complex biological process that involves the formation of new blood vessels from pre-existing ones. It plays a critical role in various physiological and pathological conditions, including the development of the corpus luteum and tumors [1,2]. Also, angiogenesis is a tightly regulated process involving endothelial cell proliferation, migration, and differentiation. Moreover, angiogenesis plays a pivotal role in physiological processes such as embryonic development, wound healing, and the menstrual cycle [10,11]. It can also contribute to the progression of diseases, particularly ovarian cancer [12].

In the corpus luteum and tumors, the balance between pro-angiogenic and anti-angiogenic factors is critical. In the corpus luteum, as the early luteal phase transitions to the mid-luteal phase, anti-angiogenic factors, such as thrombospondin-1 (TSP-1) and angiopoietin-2 (Ang-2), are upregulated. These factors help stabilize the vasculature and maintain a functional corpus luteum [13]. Similarly, in tumors, the expression of anti-angiogenic factors, including angiostatin and endostatin, can counteract the effects of pro-angiogenic factors. These anti-angiogenic factors inhibit endothelial cell proliferation and migration, maintaining a balance between angiogenic stimulators and inhibitors [14,15]. As mentioned above, the angiogenesis microenvironments of the corpus luteum and tumors share several similarities in terms of their cellular interactions, signaling molecules, and extracellular factors.

### 2.1. Angiogenesis in Corpus Luteum

In the corpus luteum, following ovulation, the ruptured follicle transforms into a structure called the corpus luteum, which requires angiogenesis to sustain its function [16]. Hypoxia within the developing corpus luteum triggers the secretion of pro-angiogenic factors, such as the vascular endothelial growth factor (VEGF) and fibroblast growth factor (FGF). These factors promote endothelial cell proliferation, migration, and tube formation, leading to the formation of new blood vessels [5,17,18]. Similarly, in tumors, rapid growth leads to an inadequate blood supply, resulting in hypoxia. This hypoxic state triggers the release of pro-angiogenic factors, primarily VEGF, by tumor cells and stroma cells. The pro-angiogenic factors promote the recruitment of new blood vessels that supply oxygen and nutrients to the growing tumor [19].

### 2.2. Angiogenesis in Tumor

Tumors, characterized by uncontrolled cell growth and division, require a dedicated blood supply to sustain their growth and survival. Angiogenesis is a critical part of the tumor microenvironment [20,21]. This process is regulated by a delicate balance between proangiogenic and antiangiogenic factors. Proangiogenic factors, such as the vascular endothelial growth factor (VEGF), the fibroblast growth factor (FGF), and the platelet-derived growth factor (PDGF), are secreted by tumor cells and stromal cells in response to hypoxia and other stimuli. These factors promote the proliferation and migration of endothelial cells. They lead to the sprouting of new blood vessels toward the tumor [12,20,22]. New blood vessels are often disorganized and leaky, further contributing to the chaotic growth of the tumor.

### 2.3. Cellular Interaction

The angiogenic microenvironment in the corpus luteum and tumors involves interactions between various cell types, including endothelial cells, pericytes, immune cells, and stromal cells. Endothelial cells are the primary cellular component of blood vessels and play a central role in angiogenesis. In both the corpus luteum and tumors, endothelial cells are stimulated to proliferate, migrate, and form new blood vessels in response to pro-angiogenic signals. Pericytes, located around endothelial cells, provide structural support to blood vessels and regulate vessel stability and maturation. They interact with endothelial cells through cell–cell contacts and secrete factors that modulate angiogenesis [23,24]. Immune cells, such as macrophages and lymphocytes, are also present in the angiogenic microenvironments of the corpus luteum and tumors. These immune cells can secrete pro-angiogenic factors, such as the vascular endothelial growth factor (VEGF) and fibroblast growth factor (FGF), to promote angiogenesis [6,25,26,27]. Alternatively, they can release anti-angiogenic factors, such as thrombospondin-1 (TSP-1) and angiostatin, to inhibit angiogenesis [6,28].

### 2.4. Signaling Molecules

Various signaling molecules play crucial roles in regulating angiogenesis in the corpus luteum and tumors. These molecules include growth factors, cytokines, chemokines, and extracellular matrix (ECM) components. In the corpus luteum, angiogenesis is tightly regulated by the balance between pro-angiogenic and anti-angiogenic factors. VEGF, FGF, and angiopoietin are secreted by luteal cells and promote endothelial cell proliferation, migration, and tube formation. These factors act through specific receptors on endothelial cells, initiating intracellular signaling pathways that drive angiogenesis [17,18,29]. Similarly, in tumors, pro-angiogenic factors are released by tumor cells and stromal cells. VEGF, in particular, is a key regulator of tumor angiogenesis. It stimulates endothelial cell proliferation and migration, and the formation of new blood vessels [30]. Other pro-angiogenic factors, such as FGF, PDGF, and angiopoietin, also contribute to tumor angiogenesis [12,18].

### 2.5. Extracellular Factors

The extracellular matrix (ECM) is a complex network of proteins and proteoglycans that provides structural support to tissues and regulates cellular functions. In both the corpus luteum and tumors, the ECM plays a crucial role in modulating angiogenesis [20]. ECM proteins, such as fibronectin, collagen, laminin, and hyaluronic acid, are present in the angiogenic microenvironments of both the corpus luteum and tumors. These proteins interact with endothelial cells and other cell types, influencing cell adhesion, migration, and signaling [31,32]. The ECM also acts as a reservoir for growth factors and cytokines, sequestering and releasing them to regulate angiogenesis. Enzymes involved in ECM remodeling, such as matrix metalloproteinases (MMPs), are upregulated during angiogenesis [33,34].

### 2.6. Cytokines and Chemokines

Cytokines and chemokines are small signaling molecules that regulate cell communication and immune responses. They can also modulate angiogenesis by influencing endothelial cell behavior and promoting the recruitment of immune cells [35]. In the corpus luteum, various cytokines and chemokines are involved in angiogenesis. Interleukin-8 (IL-8), for instance, promotes angiogenesis by stimulating endothelial cell migration and proliferation [36,37,38]. Additionally, tumor necrosis factor-alpha (TNF-α) and transforming growth factor-beta (TGF-β) can induce the expression of pro-angiogenic factors and contribute to angiogenesis in the corpus luteum [20,39]. Similarly, in tumors, cytokines and chemokines play important roles in angiogenesis. Interleukin-6 (IL-6), interleukin-1 (IL-1), and TNF-α, among others, are produced by tumor cells and stromal cells. They can stimulate endothelial cell proliferation and migration, promote the production of pro-angiogenic factors, and attract immune cells to the tumor microenvironment [26,40].

### 2.7. Signaling Pathways

#### 2.7.1. Vascular Endothelial Growth Factor (VEGF) Signaling Pathway

The VEGF pathway is one of the central signaling pathways involved in angiogenesis in both the corpus luteum and tumors. VEGF is a potent pro-angiogenic factor that stimulates endothelial cell proliferation, migration, and survival [41,42]. In the corpus luteum, luteal cells produce VEGF in response to luteinizing hormone (LH) stimulation [1,43]. VEGF binds to its receptors on endothelial cells, particularly VEGFR-2 (also known as Flk-1), triggering downstream signaling events [30,44]. This activation leads to the activation of phospholipase C gamma (PLCγ), which, in turn, generates inositol trisphosphate (IP3) and diacylglycerol (DAG). IP3 induces calcium release from the endoplasmic reticulum, activating protein kinase C (PKC). DAG, together with PKC, stimulates mitogen-activated protein kinase (MAPK) and phosphoinositide 3-kinase (PI3K) pathways [10,30,45]. The MAPK includes the extracellular signal-regulated kinase (ERK) cascade, promoting endothelial cell proliferation and migration [46].

Furthermore, VEGF also activates the PI3K pathway, activating Akt (also known as protein kinase B). Akt promotes endothelial cell survival and migration by regulating various downstream effectors, including the mammalian target of rapamycin (mTOR) pathway [44,47]. In tumors, tumor cells and stromal cells secrete VEGF to promote angiogenesis within the tumor microenvironment [48]. The binding of VEGF to its receptors on endothelial cells activates similar downstream signaling pathways involving PLCγ, IP3, DAG, PKC, MAPK, and PI3K. These signaling cascades promote endothelial cell proliferation, migration, and the formation of new blood vessels to support tumor growth.

Moreover, endocrine-gland-derived VEGF (EG-VEGF) is important in the ovarian corpus luteum and tumor [49,50,51]. EG-VEGF signaling regulates VEGFA secretion and angiogenin (ANG) mRNA and protein expression in the corpus luteum. Tumor cells also contain EG-VEGF, which is important in tumor angiogenesis. Both signaling pathways regulate MAPK activation and c-jun mRNA expression via EG-VEGF/PK-R1. Thus, EG-VEGF may induce cell proliferation in the angiogenesis signaling pathway.

#### 2.7.2. Fibroblast Growth Factor (FGF) Signaling Pathway

The FGF signaling pathway plays a critical role in angiogenesis by stimulating endothelial cell proliferation, migration, and tube formation. FGFs are a family of growth factors that bind to FGF receptors (FGFRs) on endothelial cells, initiating intracellular signaling cascades [52]. In the corpus luteum, FGFs are expressed by luteal and stromal cells [18,53]. The binding of FGFs to FGFRs on endothelial cells activates the Ras-MAPK pathway, leading to endothelial cell proliferation. The activation of the Ras-MAPK pathway results in the phosphorylation and activation of downstream effectors, including the ERK cascade. This pathway plays a crucial role in endothelial cell proliferation, migration, and survival, contributing to angiogenesis in the corpus luteum [54,55]. Additionally, FGF signaling activates the PI3K-Akt pathway, promoting endothelial cell survival, migration, and tube formation. Akt, a key mediator of this pathway, regulates multiple downstream effectors involved in angiogenesis, such as mTOR and endothelial nitric oxide synthase (eNOS) [47]. Similarly, tumor cells and stromal cells secrete FGFs to induce tumor angiogenesis. The binding of FGFs to FGFRs on endothelial cells activates Ras-MAPK and PI3K-Akt pathways, leading to endothelial cell proliferation, survival, and migration [10].

#### 2.7.3. Platelet-Derived Growth Factor (PDGF) Signaling Pathway

The PDGF signaling pathway is involved in angiogenesis by promoting endothelial cell recruitment and the pericyte stabilization of newly formed blood vessels [12,23]. Interestingly, there are similarities in the PDGF signaling pathways found in the angiogenesis of the corpus luteum and tumors. PDGFs are a family of growth factors that consist of five isoforms: PDGF-AA, -BB, -AB, -CC, and -DD. These isoforms bind to specific PDGF receptors, primarily PDGFR-β. The binding of PDGF ligands to their receptors initiates downstream signaling cascades that regulate angiogenesis [56,57,58]. The corpus luteum and tumor express PDGF ligands and receptors [39,58]. The binding of PDGF ligands to PDGFRs leads to receptor dimerization and activation. PDGF-α ligands primarily bind to PDGFR-α, while PDGF-β ligands can activate both PDGFR-α and PDGFR-β. The dimerization of PDGFRs results in the autophosphorylation of specific tyrosine residues within the receptor cytoplasmic domain. This autophosphorylation creates docking sites for various intracellular signaling molecules, initiating downstream signaling events [59,60,61].

The PI3K pathway is a crucial downstream signaling pathway activated by PDGF in both the corpus luteum and tumors. The binding of PDGF ligands to PDGFRs leads to the recruitment and activation of PI3K. Activated PI3K phosphorylates phosphatidylinositol 4,5-bisphosphate (PIP2) to generate phosphatidylinositol 3,4,5,-trisphosphate (PIP3). PIP3 acts as a second messenger and recruits proteins containing pleckstrin homology (PH) domains, such as Akt, to the plasma membrane [56,60]. Akt is subsequently phosphorylated and activated by phosphoinositide-dependent kinase 1 (PDK1) and the mammalian target of rapamycin complex 2 (mTORC2). Activated Akt promotes cell survival, migration, and proliferation, contributing to angiogenesis [62,63].

The Ras-MAPK pathway is another important downstream signaling pathway activated by PDGF in both the corpus luteum and tumors. Upon PDGF binding, activated PDGFRs recruit and activate Ras guanine nucleotide exchange factors (GEFs), leading to Ras activation. Activated Ras initiates a cascade of phosphorylation events, culminating in the activation of extracellular signal-regulated kinases (ERKs) in the MAPK pathway. Activated ERKs translocate to the nucleus and phosphorylate various transcription factors, resulting in gene expression in terms of endothelial cell proliferation, migration, and angiogenesis [57,60].

## 3. Apoptosis in Corpus Luteum and Tumor

The process of apoptosis, or programmed cell death, is a crucial mechanism for maintaining tissue homeostasis. Apoptosis eliminates unwanted or damaged cells, preventing their accumulation and potentially harmful effects. The dysregulation of apoptosis can result in various diseases [7]. In the corpus luteum, apoptosis occurs in a tightly regulated manner and is necessary for the regression of the structure. The lifespan of the corpus luteum is primarily determined by the balance between cell proliferation and apoptosis. During the early luteal phase, luteal cells proliferate and differentiate, increasing progesterone production. However, the corpus luteum undergoes regression in the late luteal phase, decreasing progesterone production [64,65]. In tumors, apoptosis can occur in two distinct pathways: the intrinsic and extrinsic pathways. The intrinsic pathway is primarily regulated by the balance between pro-apoptotic and anti-apoptotic proteins of the Bcl-2 family. In contrast, anti-apoptotic members, such as Bcl-2 and Bcl-xL, inhibit mitochondrial permeabilization and prevent apoptosis. The extrinsic pathway, also known as the death receptor pathway, is activated by binding specific ligands to death receptors on the cell surface. This triggers the recruitment of adaptor molecules and the activation of caspases, ultimately resulting in cell death [4,9,66,67].

### 3.1. Apoptosis in Corpus Luteum

In the corpus luteum, apoptosis is a key mechanism for the regression of the structure if pregnancy does not occur [65]. There is a decline in pro-survival factors, insulin-like growth factor 1 (IGF-1) and luteinizing hormone (LH), and an increase in pro-apoptotic factors, Fas ligand (FasL) and tumor necrosis factor-alpha (TNF-α). FasL and TNF-α trigger apoptotic pathways in the luteal cells [68,69,70]. The apoptosis of luteal cells is primarily regulated by the intrinsic pathway. Pro-apoptotic members of the Bcl-2 family promote mitochondrial outer membrane permeabilization, releasing cytochrome c. Cytochrome c activates caspase, the key apoptosis effector, resulting in luteal cell death [64,71]. In addition to the intrinsic pathway, the extrinsic pathway can contribute to apoptosis in the corpus luteum. FasL, a ligand that activates the death receptor pathway, is expressed in the regressing corpus luteum. FasL binding to its receptor Fas triggers the recruitment of adaptor molecules and the activation of caspases, leading to apoptosis [72].

### 3.2. Apoptosis in Tumor

In tumors, apoptosis can occur through two distinct pathways: the intrinsic and extrinsic pathways. The intrinsic pathway is primarily regulated by the balance between pro-apoptotic and anti-apoptotic members, such as Bcl-2 and Bcl-xL, which inhibit mitochondrial permeabilization and prevent apoptosis. The extrinsic pathway, also known as the death receptor pathway, is activated by binding specific ligands to death receptors on the cell surface. This triggers the recruitment of adaptor molecules and the activation of caspases, ultimately resulting in cell death. The tumor necrosis factor-alpha (TNF-α) and Fas ligand (Fas L) are ligands that can activate the extrinsic pathway [73]. In tumor development, apoptosis serves as a critical defense mechanism against cancer. When cells acquire genetic mutations that promote uncontrolled growth, apoptosis acts as a failsafe mechanism to eliminate these aberrant cells [4,67]. However, cancer cells can develop various mechanisms to evade apoptosis, allowing them to survive and propagate. One of the hallmarks of cancer is the dysregulation of the balance between cell proliferation and cell death, favoring cell survival and tumor growth. Cancer cells can upregulate anti-apoptotic proteins or downregulate pro-apoptotic proteins, thereby escaping apoptosis. Moreover, they can disrupt the signaling pathways involved in apoptosis, making themselves resistant to cell death signals [9,73].

### 3.3. Cellular Interactions

The microenvironments of apoptosis in both the corpus luteum and tumor involve intricate cellular interactions between different cell types. These interactions play critical roles in regulating apoptosis and maintaining tissue integrity. In the corpus luteum, apoptotic cell death occurs during the regression phase if pregnancy does not occur. The luteal cells undergo an apoptosis response to changes in hormonal signaling, particularly a decline in pro-survival factors such as the insulin-like growth factor 1 (IGF-1) and luteinizing hormone (LH) [3,68]. This apoptotic process is regulated by interactions between luteal cells and immune cells, including macrophages and lymphocytes. Immune cells release cytokines and apoptotic signals that trigger cell death in luteal cells [74]. Similarly, apoptosis can occur in tumors due to intrinsic or extrinsic stimuli. Tumor cells can undergo apoptosis in response to various signals, such as DNA damage, nutrient deprivation, or immune-mediated cell death. The interactions between tumor cells and immune cells, including cytotoxic T cells and natural killer (NK) cells, play a crucial role in regulating apoptosis. Immune cells can release cytotoxic molecules, such as perforin and granzymes, that induce apoptosis in tumor cells [7,75].

### 3.4. Signaling Molecules

The microenvironments of apoptosis in the corpus luteum and tumors involve the activation of specific signaling pathways and the modulation of various signaling molecules. In the corpus luteum, the regression phase is associated with increased pro-apoptotic factors, such as Fas ligand (FasL) and tumor necrosis factor-alpha (TNF-α). These factors bind to their respective receptors on luteal cells, initiating signaling cascades that lead to apoptotic cell death. The Fas/FasL pathway significantly regulates apoptosis in the corpus luteum [64,69,70]. Similarly, apoptosis can be induced in tumors through intrinsic or extrinsic pathways. Intrinsic apoptosis is triggered by intracellular signals, such as DNA damage or cellular stress, which activate pro-apoptotic proteins, including Bax and Bak. These proteins promote mitochondrial outer membrane permeabilization (MOMP), leading to the release of cytochrome c and the activation of caspases, the key effectors of apoptosis [76,77]. On the other hand, extrinsic apoptosis is initiated by binding death ligands, such as FasL- or TNF-related apoptosis-inducing ligands (TRAIL), to their corresponding death receptors on tumor cells. This binding activates caspase cascades and leads to apoptosis [78,79].

### 3.5. Extracellular Factors

The microenvironments of apoptosis in the corpus luteum and tumors involve the influence of extracellular factors and the extracellular matrix (ECM) on cell survival and death processes [80]. In the corpus luteum, the ECM undergoes remodeling during the regression phase, leading to changes in its composition and structure. The remodeling process involves the production and activation of various ECM-degrading enzymes, such as matrix metalloproteinases (MMPs). These enzymes facilitate the breakdown of the ECM and contribute to luteal cell apoptosis [81]. Additionally, cytokines and chemokines released by immune cells can modulate this [82]. Similarly, the ECM can modulate tumor apoptosis by influencing cell–ECM interactions, cellular signaling, and responses to apoptotic stimuli [83].

### 3.6. Cytokines

Cytokines are small signaling molecules that regulate cellular response and play important roles in apoptosis. They can influence cell survival, proliferation, and cell death pathways. In the microenvironments of the corpus luteum and tumors, several cytokines are involved in apoptosis. In the corpus luteum, cytokines such as tumor necrosis factor-alpha (TNF-α) and interferon-gamma (IFN-γ) have been implicated in regulating apoptosis. TNF-α can induce apoptosis in luteal cells by activating apoptotic signaling pathways, while IFN-γ can promote apoptosis by sensitizing cells to apoptotic stimuli [74,84]. Similarly, cytokines such as TNF-α, interleukin-1 (IL-1), and interferons can regulate tumor apoptosis. These cytokines can induce apoptosis directly or sensitize tumor cells to apoptotic signals, and they can also modulate the immune response, leading to immune-mediated cell death [85,86].

### 3.7. Signaling Pathways

#### 3.7.1. Tumor Suppressor Pathways

One of the key regulators of apoptosis in both the corpus luteum and tumors is tumor suppressor protein p53. P53 acts as a transcription factor and controls the expression of numerous genes involved in cell cycle arrest, DNA repair, and apoptosis. In response to various stresses, such as DNA damage, hypoxia, or oncogene activation, p53 is stabilized and activated [65,87]. Activated p53 promotes apoptosis through several mechanisms. It induces the transcription of pro-apoptotic genes, such as Bax and PUMA, which promote mitochondrial outer membrane permeabilization (MOMP) and cytochrome c release. Cytochrome c, along with apoptotic protease-activating factor 1 (Apaf-1) and procaspase-9, forms the apoptosome, leading to the activation of effector caspases and the execution of apoptosis [88,89]. In addition, the retinoblastoma (RB) pathway is another tumor suppressor pathway involved in apoptosis regulation. The RB protein regulates the cell cycle by inhibiting the activity of the E2F transcription factors. Activation of the RB pathway leads to cell cycle arrest and apoptosis induction. Dysregulation of the RB pathway can disrupt the balance between cell proliferation and apoptosis, contributing to tumorigenesis [90].

#### 3.7.2. Mitochondrial Pathway

The mitochondrial pathway is a central apoptosis mechanism shared between the corpus luteum and tumors. The Bcl-2 family of proteins, including anti-apoptotic members (Bcl-2, Bcl-XL) and pro-apoptotic members (Bax, Bak), regulate mitochondrial outer membrane permeabilization (MOMP) and cytochrome c release [76,77,91].

MOMP is a critical event in the intrinsic pathway of apoptosis. It involves the release of apoptogenic factors from the mitochondrial intermembrane space into the cytosol, triggering the activation of downstream apoptotic cascades. Pro-apoptotic Bcl-2 family members, such as Bax and Bak, promote MOMP. Upon receiving apoptotic signals, these proteins undergo conformational changes and translocate to the mitochondrial outer membrane, where they form channels or pores. This leads to the release of cytochrome c and other apoptogenic factors from the mitochondrial intermembrane space. Also, anti-apoptotic Bcl-2 family members, such as Bcl-2 and Bcl-xL, inhibit MOMP and protect cells from apoptosis. They interact with pro-apoptotic proteins, preventing their activation and translocation to the mitochondrial outer membrane. Under normal conditions, anti-apoptotic Bcl-2 family members prevent MOMP by binding to pro-apoptotic members and inhibiting their activity. In both the corpus luteum and tumors, MOMP is regulated by the balance between pro-apoptotic and anti-apoptotic members of the Bcl-2 protein family [92,93].

However, in response to apoptotic stimuli, the balance shifts towards pro-apoptotic proteins, leading to MOMP and cytochrome c release. In the corpus luteum and tumor, the release of cytochrome c triggers downstream apoptotic signaling events. Upon MOMP, cytochrome c is released from the mitochondrial intermembrane space into the cytosol [92]. Once in the cytosol, cytochrome c interacts with the apoptotic protease-activating factor 1 (Apaf-1) to form the apoptosome. The cytochrome c binds to Apaf-1, promoting the assembly of the apoptosome and the activation of caspase-9. This leads to the activation of effector caspases, such as caspase-3 and caspase-7, resulting in apoptotic cell death [94,95,96].

Moreover, caspases, a family of cysteine proteases, play a central role in the execution of apoptosis. In both the corpus luteum and tumors, caspase activation is a key event downstream of the mitochondrial signaling pathway. Activate caspase-9, formed upon apoptosome assembly, cleaves and activates effector caspases, such as caspase-3, -6, and -7 [95,96,97]. These effector caspases execute the dismantling of the cell by cleaving specific cellular substrates, leading to characteristic apoptotic changes, such as DNA fragmentation, cytoskeletal breakdown, and nuclear condensation [7].

#### 3.7.3. Death Receptor Pathway

The death receptor pathway, the extrinsic pathway, plays a significant role in apoptosis induction in both the corpus luteum and tumors. This pathway is initiated by binding death ligands, such as tumor necrosis factor-alpha (TNF-α) and Fas ligand (FasL), to their corresponding death receptors, TNFR1 and Fas, respectively. Two well-known death receptor systems are the Fas/FasL and TNF/TNFR systems [4,72]. TNFR1 is a death receptor that binds to TNF-α, a key death ligand. TNF-α is a cytokine involved in various physiological and pathological processes, including apoptosis regulation. The binding of TNF-α to TNF1 initiates the formation of a death-inducing signaling complex (DISC), which leads to apoptosis induction [98]. Fas, also known as CD95 or Apo-1, is another death receptor involved in apoptosis. FasL, the ligand for Fas, is expressed on the surface of cytotoxic T cells and natural killer (NK) cells. FasL binding to Fas triggers the assembly of the DISC, leading to apoptosis activation [98,99,100].

The DISC is a critical component of death receptor signaling pathways and is formed upon ligand–receptor interactions. The DISC recruits and activates caspases, the key executioners of apoptosis. Specialized adaptor proteins, such as the Fas-associated death domain protein (FADD) and TNF receptor-associated death domain (TRADD), are recruited upon ligand binding to death receptors. These adaptors, in turn, recruit and activate procaspase-8 or procaspase-10, forming the DISC [66,100].

Caspases are cysteine proteases that play a central role in the execution of apoptosis. In both the corpus luteum and tumors, the activation of caspases is a key event downstream of death receptor signaling pathways. Upon activation, procaspase-8 or procaspase-10 within the DISC undergo autoproteolytic cleavage, resulting in the formation of active caspase-8 or caspase-10. These activated caspases can directly cleave and activate downstream effector caspases, such as caspase-3, -6, and -7. The activation of effector caspases is a crucial step in the execution phase of apoptosis. Once activated, effector caspases cleave specific cellular substrates, leading to characteristic apoptotic changes [66,97]. Effector caspases cleave various cellular proteins, including nuclear lamins, DNA repair enzymes, and cytoskeletal components. These cleavages result in nuclear fragmentation, DNA degradation, and cytoskeletal breakdown, ultimately leading to cell shrinkage and fragmentation [7,97].

## 4. Conclusions

**Figure 2:**
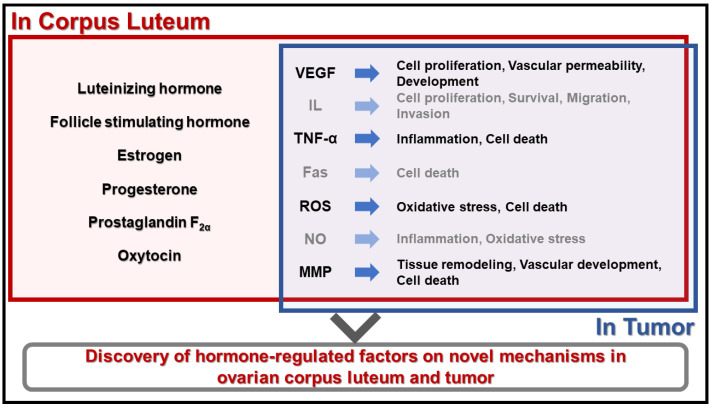
Diagram of the novel model of the hormone-regulated factors in the physiology function of the ovarian corpus luteum and tumors. Hormones play a critical role in the formation and regression of the corpus luteum by regulating several factors. This model shows a new research area that applies the mechanisms of hormones in tumor physiology. VEGF: vascular endothelial growth factor; IL: interleukin; TNF-α: tumor necrosis factor-α; ROS: reactive oxygen species; NO: nitric oxide; MMP: matrix metalloproteinase.

Apoptosis and angiogenesis are fundamental processes that play critical roles in the development, maintenance, and regression of various tissues and organs. The corpus luteum is a transient endocrine gland formed in the ovary after ovulation, and tumors are abnormal growths of cells. Both tissues undergo apoptosis and angiogenesis to fulfill their physiological functions in animals. We deduced that reproductive hormones may regulate the angiogenesis and apoptosis of tumors, since they have important functions in the corpus luteum (Figure 2). Thus, we suggest that these studies can provide valuable insights into the shared mechanisms and interactions within angiogenesis and apoptosis processes in the ovary, leading to potential physiologic targets and strategies for various ovarian diseases, including reproductive hormone functions in animal ovaries.

## Data Availability

No new data were created or analyzed in this study. Data sharing is not applicable to this article.
